# Population Pharmacokinetic Models at the Forefront of Precision Dosing

**DOI:** 10.3390/pharmaceutics18050535

**Published:** 2026-04-28

**Authors:** Katarina M. Vučićević

**Affiliations:** Department of Pharmacokinetics and Clinical Pharmacy, Faculty of Pharmacy, University of Belgrade, 11221 Belgrade, Serbia; katarina.vucicevic@pharmacy.bg.ac.rs

## 1. Population Pharmacokinetics in Focus

Population pharmacokinetics (PopPK) has evolved from a methodological niche into a fundamental pillar of quantitative clinical pharmacology, playing a key role in dose optimization and advancements in individualized therapy and regulatory decision making. By integrating compartmental pharmacokinetic models with nonlinear mixed-effects (NLME) statistical frameworks, PopPK enables the characterization of drug concentration–time profiles and the quantification of variability in drug exposure across individuals within a target population. Interindividual variability is typically explained by identifiable covariates, including demographic, physiological, and disease-related factors such as age, body size, organ function, and underlying clinical conditions [1,2,3]. Through this framework, PopPK analyses enable us to make better interpretations of clinical observations, guide evidence-based dose adjustments, and support decision making across diverse therapeutic areas. In recent decades, continuous advancements in modeling methodologies, together with increased computational capacity, and the growing availability of high-quality clinical data have substantially strengthened the application of PopPK in both drug development and clinical practice [4,5].

Recent reviews emphasize that model-informed precision dosing (MIPD) represents the next generation of personalized therapy, extending beyond conventional therapeutic drug monitoring (TDM) to incorporate patient-specific characteristics, longitudinal measurements, and predictive modeling, thereby improving the precision of dosing recommendations. Notably, Minichmayr and colleagues demonstrated how MIPD leverages pharmacometric models to facilitate the routine clinical implementation of model-informed approaches, thereby advancing individualized therapy across diverse therapeutic areas, including infectious diseases, oncology, and inflammatory disorders [6].

The Special Issue of *Pharmaceutics* titled “Population Pharmacokinetics and Its Clinical Applications, 2nd edition” built upon the first collection published in 2024, which showcased recent advances in both the development and application of PopPK models across clinical contexts and special populations, highlighting their role in MIPD-TDM, and individualized therapy.

## 2. Highlights of the Special Issue

The collection of manuscripts published in this Special Issue of *Pharmaceutics*—“Population Pharmacokinetics and Its Clinical Applications”—reflects a coherent and timely advancement in the field of pharmacometrics, with a particular emphasis on PopPK models and their clinical application. Despite the diversity of therapeutic areas, including oncology, neurology, transplantation, inflammatory and rare diseases, the contributions converge on a shared objective: translating quantitative models into clinically actionable tools that support individualized therapy.

A central theme across the Special Issue is the development and refinement of PopPK models as a foundation for understanding drug disposition and variability. Several studies, including those on gabapentin (Contribution 8), pamiparib (Contribution 13), tideglusib (Contribution 14), and cyclosporin A (Contribution 15), systematically characterize drug absorption, distribution, metabolism, and elimination using an NLME modeling approach. These analyses consistently highlight the role of patient-specific covariates, such as renal function, body weight, age, hemoglobin levels, and prior therapies in explaining interindividual variability in exposure. In rare diseases, the development of a PopPK model for tideglusib demonstrates how modeling approaches can support pediatric extrapolation and inform dosing strategies in conditions with limited clinical data (Contribution 14).

Beyond PopPK model development, several manuscripts extend into PK/PD modeling, linking drug exposure to clinical outcomes. The study of palbociclib in pediatric patients with brain tumor exemplifies this integration by connecting exposure to hematological toxicity endpoints, enabling simulation-based risk assessment (Contribution 9). Similarly, the investigation of vedolizumab in inflammatory bowel disease demonstrates how drug clearance may outperform traditional exposure metric (trough concentration) as predictors of clinical remission (Contribution 11). These findings underscore a paradigm shift toward more mechanistically meaningful biomarkers derived from models rather than empirical thresholds alone.

A distinguishing feature of this Special Issue is its strong focus on MIPD as a bridge between pharmacometric theory and clinical decision making. The paired studies on ustekinumab (Contribution 6) and secukinumab (Contribution 10) in psoriasis provide compelling examples of how PK/PD models can be operationalized to individualize dosing regimens. By explicitly accounting for uncertainty in individual parameter estimates, these works move beyond deterministic predictions and introduce probabilistic frameworks that better reflect clinical reality. Notably, both studies demonstrate that a substantial proportion of patients may benefit from dose optimization or intensification compared to standard regimens, highlighting the clinical impact of MIPD strategies.

Complementing these efforts are contributions that emphasize translational tools and implementation. The development of a web-based application for simulating zolpidem concentrations in intoxication scenarios represents a practical step toward bedside application of PopPK models (Contribution 2). Similarly, the use of simulation platforms in studies such as cyclosporin A (Contribution 15) further illustrates how model outputs can be directly integrated into clinical workflows. These approaches align with the broader vision of digital health and decision-support systems, where complex models are embedded into user-friendly interfaces accessible to clinicians.

Methodologically, the Special Issue also showcases innovation in model design. The semi-physiological model of clopidogrel incorporating first-pass metabolism and metabolite formation highlights the value of mechanistic modeling in capturing complex biotransformation processes (Contribution 1). Meanwhile, the joint modeling of total and unbound drug concentrations, as demonstrated for pamiparib, reflects an increasing recognition of the importance of pharmacologically active fractions in dose optimization (Contribution 13).

The manuscripts included in the Special Issue collectively demonstrate that PopPK has evolved beyond a purely theoretical framework into a clinically applicable discipline that underpins precision medicine in real-world settings. Together, these contributions illustrate the full continuum of PopPK model application: from data generation and rigorous model development using NLME approaches, through model evaluation and simulation, to clinical translation and implementation in decision-support contexts. In this context, the manuscripts complement a growing body of evidence supporting MIPD as an effective framework for optimizing pharmacotherapy.

## 3. Towards Precision Pharmacology

The field of PopPK is poised for continued transformation as technological advances, computational methods, and clinical integration converge. One of the most promising directions is selection of the adequate PopPK model, and the broader implementation of MIPD in routine clinical practice. This will require not only robust PopPK models but also user-friendly software platforms, integration with electronic health records, and real-time analytics to support clinicians in making data-driven dosing decisions [6]. Nevertheless, integrating biomarkers and pharmacodynamic endpoints with PopPK models will strengthen individualized therapy by linking drug exposure to clinical outcomes. Prospective clinical trials validating these approaches will be essential to translate the promise of MIPD into measurable improvements in patient care, safety, and efficacy.

Artificial intelligence and machine learning are expected to complement traditional population models by identifying hidden covariates, predicting drug response patterns, and integrating complex datasets from genomics, metabolomics, and patient monitoring devices. Such approaches could refine model predictions and extend MIPD to complex multi-drug regimens, especially in oncology, infectious diseases, and transplant medicine [7,8,9].

In summary, the future of PopPK lies in its full integration into precision medicine, supported by technology, enriched datasets, and validated clinical outcomes. Continued collaboration among modelers, clinicians, regulatory authorities, and software developers will be key to realizing the transformative potential of MIPD in improving patient-centered therapy.
